# Monoclonal gammopathy of renal significance (MGRS)-related AL amyloidosis complicated by amyloid myopathy: a case report

**DOI:** 10.1186/s12882-021-02272-7

**Published:** 2021-02-27

**Authors:** Erina Ono, Akira Ishii, Yoshiaki Higashi, Natsuko Koita, Takashi Ayaki, Katsuya Tanigaki, Shunsuke Takayanagi, Naoya Kondo, Kaoru Sakai, Shuichiro Endo, Hideki Yokoi, Takeshi Matsubara, Sachiko Minamiguchi, Ichizo Nishino, Ryosuke Takahashi, Motoko Yanagita

**Affiliations:** 1 Department of Nephrology, Graduate School of Medicine, Kyoto University, 54 Shogoin Kawahara-cho, Sakyo-ku, Kyoto, 606-8507 Japan; 2 Department of Neurology, Graduate School of Medicine, Kyoto University, 54 Shogoin-Kawahara-cho, Sakyo-ku, Kyoto, 606-8507 Japan; 3 Department of Diagnostic Pathology, Graduate School of Medicine, Kyoto University, 54 Shogoin-Kawahara-cho, Sakyo-ku, Kyoto, 606-8507 Japan; 4 Department of Neuromuscular Research, National Institute of Neuroscience, National Center of Neurology and Psychiatry (NCNP), 4-1-1 Ogawa-Higashi, Kodaira, Tokyo, 187-8502 Japan

**Keywords:** Amyloidosis, Amyloid myopathy, Monoclonal gammopathy of undetermined significance, Monoclonal gammopathy of renal significance, Sporadic late-onset nemaline myopathy

## Abstract

**Background:**

Lately, monoclonal gammopathy of renal significance (MGRS) has been defined as a group of renal disorders that are strongly associated with monoclonal protein, including amyloid immunoglobulin light chain (AL) amyloidosis. Amyloid myopathy is rare (1.5% of all patients with amyloidosis) and the prognosis is poor. Furthermore, only approximately 20% of patients with amyloid myopathy are reported to have renal involvement, indicating a lack of data in the literature.

**Case presentation:**

Here, we report a rare case of MGRS-related AL amyloidosis complicated by amyloid myopathy that presented with muscle weakness in the upper and lower limbs, neck and fingers, and nephrotic syndrome. Blood, urine, and bone marrow examination revealed monoclonal gammopathy of undetermined significance (MGUS) (Bence Jones protein-lambda). Muscle biopsy of the vastus lateralis muscle demonstrated amyloid proteins in the sarcolemma and in the blood vessel walls on Congo red staining, suggesting amyloid myopathy, and tiny inclusions in fibers on modified Gomori trichrome stain. Although we thought they were reminiscent of nemaline bodies, we could not confirm the nature of this structure. Renal biopsy demonstrated amyloid proteins in the mesangial region, part of the capillary walls, and the blood vessel walls on direct fast scarlet staining. As these amyloid proteins were positive for p-component staining and negative for amyloid A staining, β2-microglobulin, and pre-albumin, and as lambda light chains were positive in the mesangial region, we diagnosed the patient with MGRS-related AL amyloidosis. Although he was treated with melphalan and dexamethasone, his symptoms did not improve.

**Conclusions:**

AL amyloidosis involving the kidneys and muscles has a poor prognosis, and a delayed diagnosis of amyloid myopathy is common because of its rarity and frequent misdiagnosis, which increases organ function deterioration. Therefore, early detection, therapeutic intervention, and careful follow-up are crucial.

## Background

Monoclonal gammopathy of renal significance (MGRS) has been defined in recent years as a group of renal disorders that are strongly associated with monoclonal protein (M protein), including amyloid immunoglobulin light chain (AL) amyloidosis, light-chain deposition disease, immunotactoid glomerulonephritis, and proliferative glomerulonephritis with monoclonal Ig deposits (PGNMID) [1–3]. The concept of MGRS is attracting attention because the significance of early treatment for MGRS-related kidney disease targeting the responsible small B-cell clones has been reported in terms of preventing renal deterioration [4]. AL amyloidosis is characterized by the presence of monoclonal protein and amyloid deposits in various organs [5, 6]. Although involvement of the kidney, heart, liver, and nervous system commonly occurs in systemic amyloidosis [5], it has been reported that the complication of amyloid myopathy is rare (1.5%) and the prognosis is poor (approximately 22–32 months from onset of symptoms to death) [7–9]. Furthermore, only 16 to 25% of patients with amyloid myopathy are reported to have renal involvement [7, 10]. There is a scarcity of data on amyloid myopathy with nephropathy in the scientific literature. Nemaline myopathy is a group of muscle diseases pathologically characterized by the presence of nemaline rods in myofibers on modified Gomori trichrome stain. Sporadic late-onset nemaline myopathy (SLONM) affects adults in the middle age to elderly period and involves subacutely progressive weakness and atrophy in the axial and predominantly proximal limb muscles [11]. Approximately half of SLONM patients have concomitant monoclonal gammopathy of undetermined significance (MGUS), and the prognosis of SLONM with MGUS (SLONM-MGUS) is reported to be poor mainly due to respiratory failure, leading to death usually within a few years [12].

Here, we report a rare case of MGRS-related AL amyloidosis complicated by amyloid myopathy detected by upper and lower limb, neck flexion and extension, flexor and extensor digitorum muscle weakness, and nephrotic syndrome.

## Case presentation

An 83-year-old man had a two-year history of proteinuria (2+) that had become worse in the year before admission. He complained of progressive proximal lower limb muscle weakness for 10 months before admission. He was admitted to our hospital because of upper and lower limb, neck flexor and extensor, and flexor and extensor digitorum muscle weakness, and pitting edema of the legs. He had a medical history of atrial fibrillation and hypertension. He had smoked five cigarettes per day for the decade before he turned 30.

**Fig. 1:**
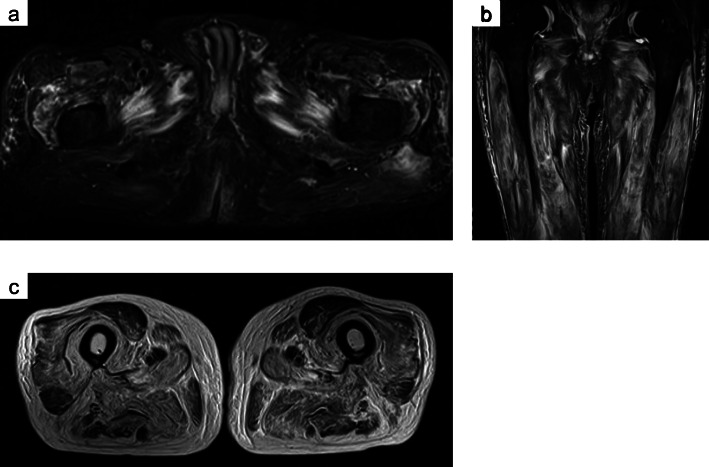
Muscle magnetic resonance imaging scan images. Axial (**a**) and coronal (**b**) short inversion time inversion recovery (STIR) images and axial T2 weighted image (**c**) show muscle atrophy and high intensity signals in the quadriceps femoris, obturator, and gluteus maximus muscles

On admission, predominantly proximal symmetrical muscle weakness and leg pitting edema were observed. His manual muscle testing (MMT) scores were: neck flexion = 4; neck extension = 4; deltoids = 3; biceps brachii = 3; triceps brachii = 3; flexor and extensor muscles of the hand = 4; flexor and extensor digitorum muscle = 3; iliopsoas = 2; gluteus maximus = 2; quadriceps = 2; hamstring = 2; flexor and extensor muscles of the foot = 5. His blood pressure was 120/80 mmHg. Laboratory tests on admission showed serum creatinine and albumin levels of 0.7 mg/dL and 2.9 g/dL, respectively, and his urinary protein creatinine ratio was 7.3 g/gCr, suggesting nephrotic syndrome. Serum creatine kinase (820 U/L), aldolase (13.7 IU/L), and urinary myoglobin levels were initially elevated, but after admission they normalized and myoglobinuria disappeared. All autoantibodies (i.e., antinuclear antibodies [ANA], anti-double stranded DNA [anti-dsDNA] antibodies, anti-Ro/SS-A antibodies, anti-La/SS-B antibodies, anti-aminoacyl tRNA synthetase [anti-ARS] antibodies, anti-cyclic citrullinated peptide [anti-CCP] antibodies, anti-mitochondrial M2 [AMA2] antibodies, acetylcholine receptor [AChR] antibodies, and anti-muscle-specific kinase [anti-MuSK] antibodies) were negative. Although serum immunoglobulin (Ig) G was decreased (705 mg/dL), IgA (159 mg/dL) and IgM (37 mg/dL) were within normal range. Bence Jones protein (BJP)-lambda was observed in both the blood and urine, the serum free light chain lambda was 533 mg/L, the serum kappa/lambda ratio was 0.046, and the M protein level was 0.575 g/dL. The plasma cell count in the bone marrow was 0.7%, indicating MGUS. Magnetic resonance imaging (MRI) scan showed proximal lower limb muscle atrophy and edema, including the quadriceps femoris, obturator, and gluteus maximus muscles (Fig. 1). Needle electromyogram was performed in the first dorsal interosseous, deltoid, and iliopsoas muscles. Although it showed fibrillation potential and positive sharp waves, it also showed complex repetitive discharge, low amplitude, and early recruitment, suggestive of myogenic changes. Nerve conduction study of the median, ulnar, tibial, and sural nerves was normal (Table 1). Therefore, the patient was diagnosed with myopathy as the cause of the predominantly proximal symmetrical muscle weakness.

**Table 1:** Nerve conduction study

Motor nerves/site	Amplitude (mV)	Latency (ms)	NCV (m/s)	F-wave minimal latency (ms)
Median nerve				
Wrist	5.7	3.6		25.1
Elbow	5.4	7.2	54.9	
Ulnar nerve				
Wrist	6.1	2.8		24.8
Below elbow	5.9	5.8	65.1	
Above elbow	5.7	7.1	56	
Tibial nerve				
Ankle	4.6	3.8	44.9	44.8
Popliteal fossa	3.3	11.8		
Sensory nerves	Amplitude (μV)	Latency (ms)	NCV (m/s)	
Median nerve	24.4	2.8	57	
Ulnar nerve	16.1	2.3	60	
Sural nerve	Not evoked (*due to leg edema)			

*NCV* indicates nerve conduction velocity

**Fig. 2:**
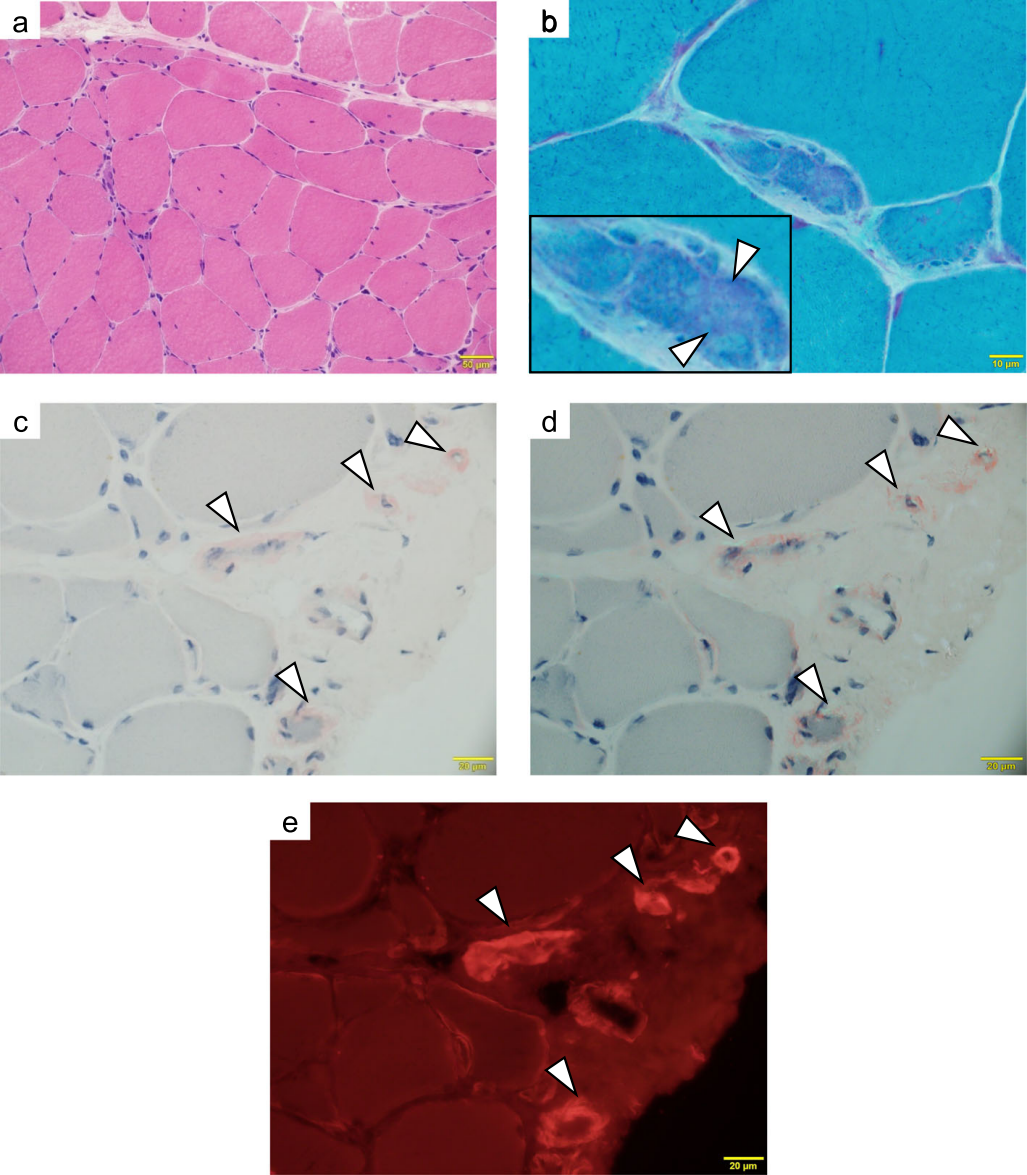
Muscle biopsy specimen on light microscopy. Hematoxylin and eosin staining (**a**) shows a small cluster of atrophic angulated fibers and mild fiber size variation. Modified Gomori trichrome stain (**b**) shows tiny inclusions in fibers (white arrowheads). Congo red staining (**c**) shows amyloid proteins in which *birefringence* is observed under the polarizing microscope (**d**) and which is positive under the fluorescence microscope (**e**) in the sarcolemma and blood vessel walls (white arrowheads)

**Fig. 3:**
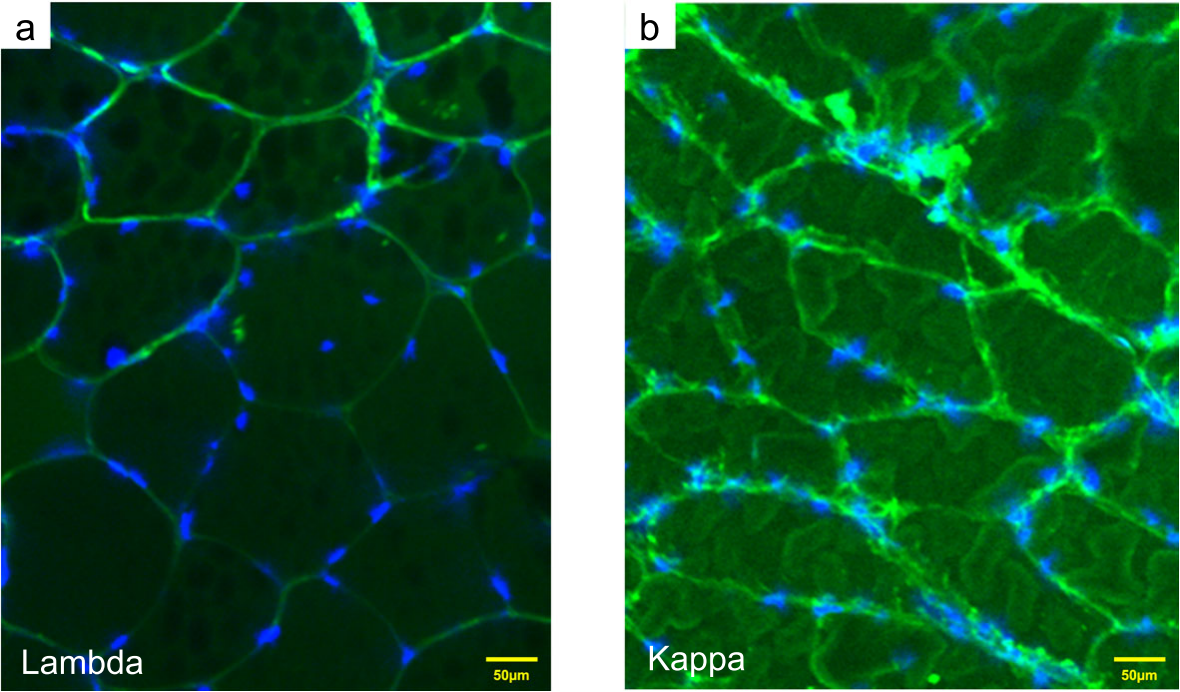
Muscle biopsy specimen on immunofluorescence microscopy. Blue indicates 4′,6-diamidino-2-phenylindole and green indicates lambda (**a**) and kappa (**b**), both of which are negative

A muscle biopsy of the right vastus lateralis muscle was performed on the 25th day of admission. Light microscopy showed a small cluster of atrophic angulated fibers in addition to mild fiber size variation on hematoxylin and eosin (HE)–stained sections. On nicotinamide adenine dinucleotide-tetrazolium reductase (NADH-TR), some fibers minimally lacked oxidative activity (Fig. 2a). On modified Gomori trichrome, one atrophic fiber was filled with fine cytoplasmic inclusions (Fig. 2b). Although we thought they were reminiscent of nemaline bodies, we could not confirm the nature of this structure as a sample for electron microscopic study was not available. Congo red positivity in the sarcolemma membrane and vascular walls was observed (Fig. 2c, d, e). Immunofluorescence staining of frozen muscle tissue showed that lambda and kappa light chains were negative (Fig. 3). Considering the clinical course and histopathological findings, the patient was diagnosed with amyloid myopathy.

**Fig. 4:**
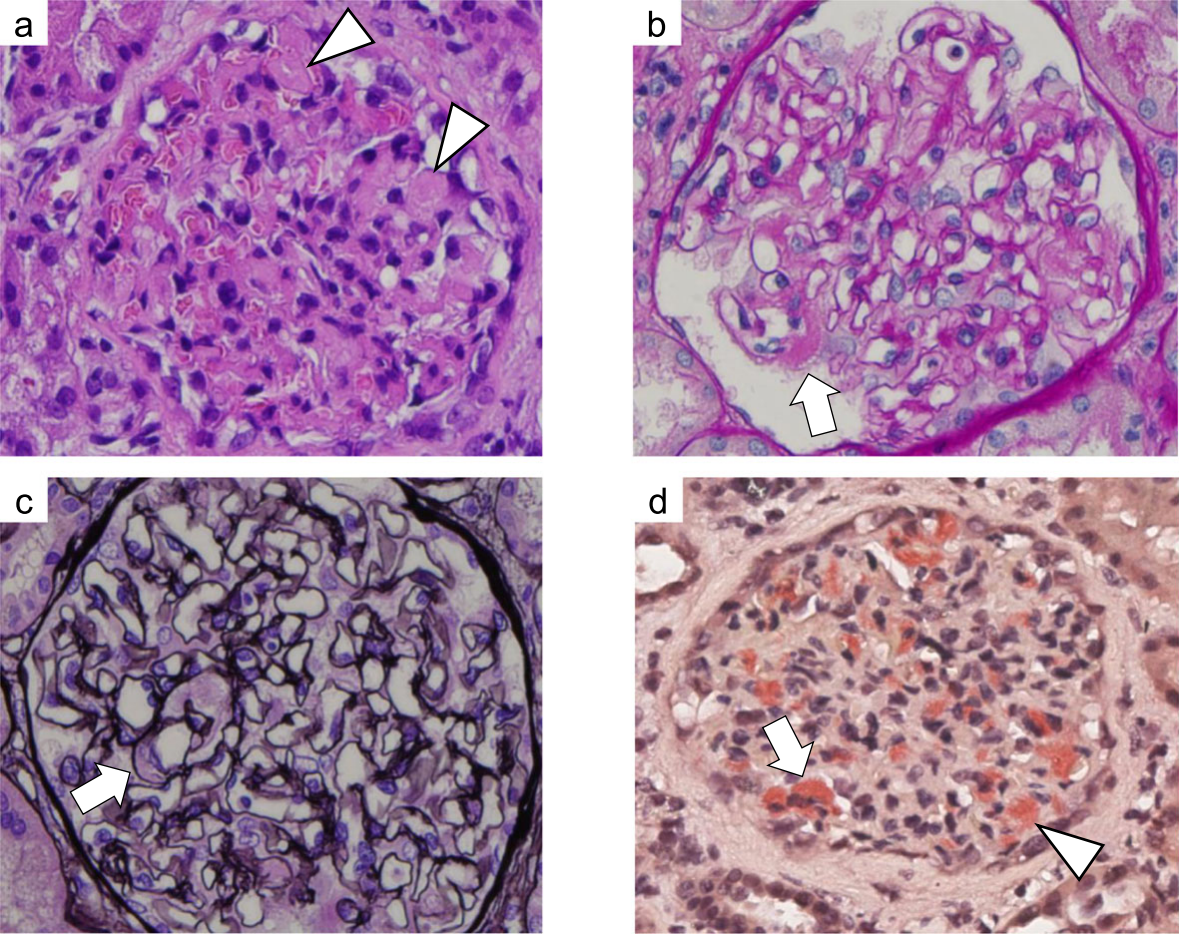
Renal biopsy specimen on light microscopy. Hematoxylin and eosin staining (**a**; × 400), periodic acid–Schiff staining (**b**; × 400), and periodic acid–methenamine-silver staining (**c**; × 400) in light microscopy show an accumulation of eosinophilic and amorphous deposits in the mesangial region (white arrowheads) and part of the capillary walls (white arrows). Direct fast scarlet staining (Panel d; × 400) shows amyloid proteins in the mesangial region (white arrowhead) and part of the capillary walls (white arrow)

**Fig. 5:**
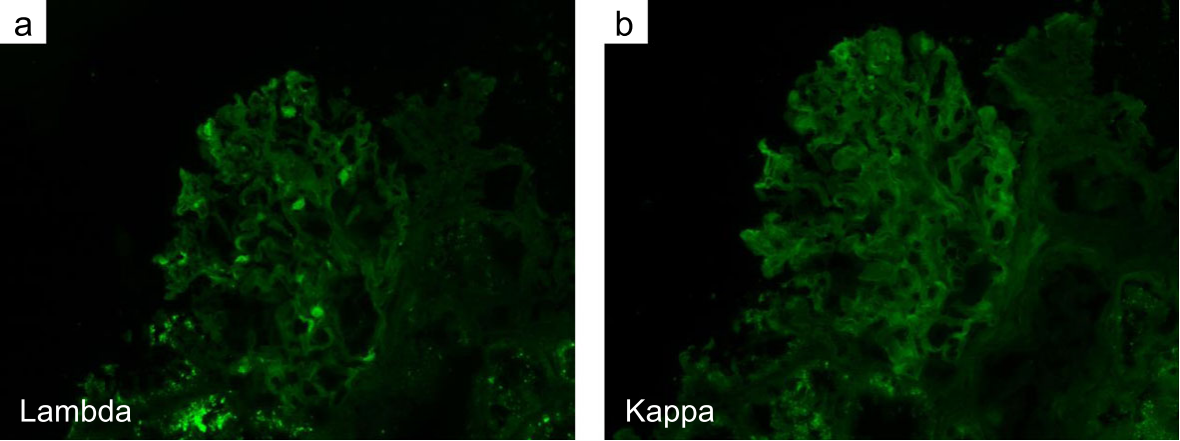
Renal biopsy specimen on immunofluorescence microscopy. Direct immunofluorescence microscopy shows (1+) staining for lambda light chains (**a**; × 200) in the mesangial region and negative staining for kappa light chains (**b**; × 200)

**Fig. 6:**
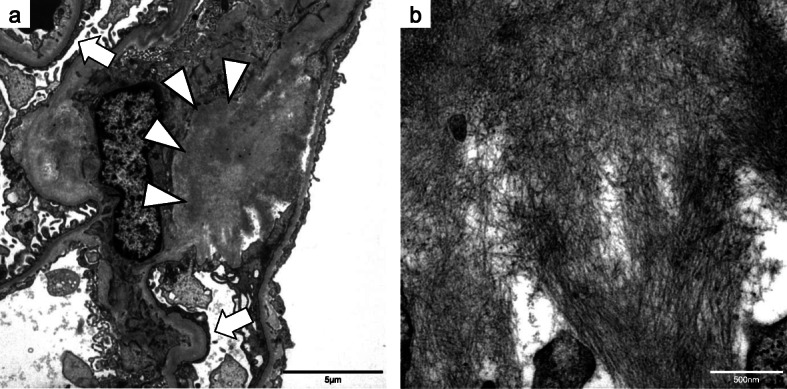
Renal biopsy specimen on electron microscopy. Electron microscopy (**a**; × 4000, **b**; × 30,000) shows amyloid fibrils in the mesangial region (white arrowheads) and podocyte foot process effacement (white arrows). The fiber diameter is between 9 nm and 12 nm

A percutaneous renal biopsy was performed on the 35th day of admission to determine the cause of nephrotic syndrome. Light microscopy showed an accumulation of eosinophilic, amorphous deposits in the mesangial region and part of the capillary walls in HE-stained sections (Fig. 4a), periodic acid–Schiff–stained sections (Fig. 4b), and periodic acid–methenamine-silver–stained sections (Fig. 4c), which were determined to be amyloid proteins as they were stained positive by direct fast scarlet staining (Fig. 4d). Amyloid proteins were also observed in the blood vessel walls. As these amyloid proteins were positive for p-component staining and negative for amyloid A staining, β2-microglobulin, and pre-albumin, we diagnosed the patient with AL amyloidosis. Approximately 40% interstitial fibrosis and tubular atrophy was observed. Immunofluorescence staining of frozen tissue showed that lambda light chains were positive in the mesangial region (Fig. 5a). Staining with IgG, IgA, IgM, complement component 3 (C3)c, C1q, and kappa light chains were negative (Fig. 5b). Electron microscopy showed amyloid fibrils in the mesangial region (Fig. 6). Thus, the patient was diagnosed with AL amyloidosis caused by BJP-lambda.

Each examination was subsequently performed for systemic amyloidosis. Although echocardiography showed mild left ventricular diastolic dysfunction without characteristic findings for cardiac amyloidosis such as a classical granular sparkling appearance, we did not exclude cardiac amyloidosis because 1) both serum NT-pro brain natriuretic peptide (BNP) (369.7 pg/mL) and troponin T levels (0.166 ng/mL) were elevated and 2) the patient had a history of atrial fibrillation. Amyloid protein was negative in the colon biopsy. Finally, the patient was diagnosed with AL amyloidosis involving the kidneys, muscles, and possibly the heart.

The patient was treated with MD (melphalan, 8 mg and dexamethasone, 20 mg daily for 4 days) therapy for AL amyloidosis. Although his cardiac function and respiratory function did not decline, the muscle weakness grew worse, and the nephrotic syndrome, renal insufficiency, and serum free light chain level did not improve. Twenty-one months after therapy commenced, the serum creatinine level had increased to 1.2 mg/dL, and his MMT scores were: neck flexion = 3; neck extension = 3; deltoids = 2–3; biceps brachii = 2–3; triceps brachii = 2–3; flexor and extensor muscles of the hand = 2–3; flexor and extensor digitorum muscle = 2–3; iliopsoas = 1–2; gluteus maximus = 1–2; quadriceps = 1–2; hamstring = 1–2; flexor and extensor muscles of the foot = 2–3.

## Discussion and conclusions

Here, we report a rare case of drug-resistant MGRS-related AL amyloidosis complicated by amyloid myopathy.

We could identify the cause of the muscle weakness and nephrotic syndrome through muscle and renal biopsies. The complication of amyloid myopathy is rare. In previous reports, only 1.5% of AL amyloidosis patients had amyloid myopathy, and only 16 to 25% of those with amyloid myopathy had renal involvement [7, 10]; the percentage of men was 71% and the median age was 67 years [7]. Common symptoms of amyloid myopathy include predominantly proximal muscle weakness, muscle pseudohypertrophy, muscle atrophy, exercise-induced myalgia, macroglossia, and dysphagia [7, 8, 10]. There are three main clinical presentation patterns: 1) skeletal pseudohypertrophy with macroglossia and palpable nodules in the muscles; 2) muscle weakness and atrophy only without other signs such as pseudohypertrophy or macroglossia; and 3) a combination of these two forms [7–10]. This elderly male patient presented with predominantly proximal symmetrical muscle weakness and muscle atrophy, which are common in amyloid myopathy, and muscle weakness of the neck and fingers, which is not common in amyloid myopathy. He had the second type of clinical presentation pattern, which has been reported to be particularly difficult to diagnose because the symptoms are nonspecific and may mimic those of polymyositis and primary inflammatory rheumatic diseases [7–9, 13, 14]. The electrophysiologic and pathological findings are also nonspecific, except for amyloid positivity [7–9, 14]. Because of its rarity and frequent misdiagnosis due to nonspecific presentations, a delayed diagnosis of the complication of amyloid myopathy is common (median time from first symptom to diagnosis is 17.5–23 months) [7, 9, 10]. Hematologic response is an essential prerequisite for maintaining or improving organ functions and prolonging survival. A delay in chemotherapy initiation (and consequently in hematologic response) results in further deposition of amyloid that reduces the chances of improvement of organs, suggesting that early diagnosis and therapeutic intervention are important [1, 7, 9, 15]. In order to diagnose amyloid myopathy without delay, confirming the presence of monoclonal protein in the blood or urine and amyloid positivity in muscle biopsy is important [8]. The percentage of multiple myeloma in AL amyloidosis was only 15% and even MGUS could cause AL amyloidosis [4]. In this case, the time from onset of muscle weakness to diagnosis of amyloid myopathy was only 10 months because we suspected the complication of amyloid myopathy due to the presence of MGUS (BJP-lambda) and AL amyloidosis detected by renal biopsy. We diagnosed this patient with amyloid myopathy based on the Congo red positivity with birefringence in muscle biopsy (electron microscopic study was not available). Although lambda light chains were positive in renal biopsy, they were negative in muscle biopsy. We believe that this may be related to technical problems and limitations in light chain staining in muscle biopsy. Only a few reports have performed light chain staining in muscle biopsy [8, 14, 16, 17], and further research is thus necessary. Although we considered fine cytoplasmic inclusions, which were observed in one atrophic fiber on modified Gomori trichrome stain in muscle biopsy, were reminiscent of nemaline bodies, we could not confirm the nature of this structure and did not diagnose the patient with SLONM. However, if patients with MGRS including AL amyloidosis present with muscle weakness of the neck and fingers, we consider it important to include SLONM in the differential diagnoses. The reasons are as follows: 1) this symptom is reported to be relatively rare in amyloid myopathy [7, 8, 10] but common in SLONM [18–21]; 2) half of SLONM patients were reported to have MGUS [12, 18]; and 3) the prognosis of SLONM-MGUS is poor, but certain treatments were reported to be effective in some cases [12, 18, 21–23].

The standard first-line treatment of AL amyloidosis is high-dose melphalan followed by autologous peripheral blood stem cell transplantation (HDM-SCT) [5]. For those patients who are ineligible for transplantation, second-line treatment such as cyclophosphamide, bortezomib, and dexamethasone (CyBorD) or third-line treatment such as melphalan and dexamethasone (MD) are recommended [5]. In this case, the patient was ineligible for HDM-SCT because of age, performance status, serum troponin T level, and a history of multiple organ failures of the kidneys, muscles, and heart. Furthermore, CyBorD, which has a side effect of cardiotoxicity, was also ineligible because the patient had a possible cardiac amyloidosis complication. We thus started MD treatment but neither the muscle weakness nor nephrotic syndrome improved. Although it was reported that MD was the most common treatment in amyloid myopathy and improved survival in patients whose survival was less than 8 years, only 8 of 23 patients with amyloid myopathy responded to treatment, including other therapy [7, 8, 24]. Daratumumab, pomalidomide, and doxycycline are promising agents for refractory AL amyloidosis [5, 24]. We are also considering adding another therapy in the future.

The prognostic factor of AL amyloidosis is the complication of cardiac involvement, and the median survival duration was reported to be 55 months in Revised Mayo stage 1, 19 months in stage 2, 12 months in stage 3, and 5 months in stage 4. This case was classified as Revised Mayo stage 3 based on the serum troponin T level (≥0.025 ng/mL) and the difference between the involved and uninvolved serum free light chain (≥180 mg/L) [6]. The prognosis of renal amyloidosis or amyloid myopathy is also poor (approximately 22–32 months from onset of symptoms due to amyloid myopathy to death) [7, 8, 25]. This patient lived for at least 21 months despite the fact that he had no improvement in symptoms. In contrast, the prognostic factor of SLONM is the complication of MGUS, and the majority of SLONM-MGUS patients are reported to die from respiratory failure within a few years [12]. In the future, in cases in which the complication of SLONM is suspected, we should carefully monitor the patients for both cardiac dysfunction due to the progression of cardiac amyloidosis and respiratory failure due to the progression of respiratory muscle atrophy.

In summary, we report a rare case of MGRS-related AL amyloidosis complicated by amyloid myopathy. AL amyloidosis involving the kidneys and muscles has a poor prognosis, and a delayed diagnosis of amyloid myopathy is common because of its rarity and frequent misdiagnosis, which increases organ function deterioration. Therefore, early detection, therapeutic intervention, and careful follow-up are crucial.

## Data Availability

The dataset supporting the conclusions of this article is included within the article.

## Abbreviations

AChR: Acetylcholine receptor
AL: Amyloid immunoglobulin light chain
AMA2: Anti-mitochondrial M2
ANA: Antinuclear antibodies
anti-ARS: Anti-aminoacyl tRNA synthetase
anti-CCP: Anti-cyclic citrullinated peptide
anti-dsDNA: Anti-double-stranded DNA
anti-MuSK: Anti-muscle-specific kinase
BNP: Brain natriuretic peptide
BJP: Bence Jones protein
C3: Complement component 3
CyBorD: Cyclophosphamide, bortezomib, and dexamethasone
HDM-SCT: High-dose melphalan followed by autologous peripheral blood stem cell transplantation
HE: Hematoxylin and eosin
Ig: Immunoglobulin
MD: Melphalan and dexamethasone
MGRS: Monoclonal gammopathy of renal significance
MGUS: Monoclonal gammopathy of undetermined significance
MMT: Manual muscle testing
M protein: Monoclonal protein
MRI: Magnetic resonance imaging
NADH-TR: Nicotinamide adenine dinucleotide-tetrazolium reductase
PGNMID: Proliferative glomerulonephritis with monoclonal Ig deposits
SLONM: Sporadic late-onset nemaline myopathy
SLONM-MGUS: SLONM with MGUS
STIR: Short inversion time inversion recovery
